# “Walking on eggshells”: experiences of underrepresented women in medical training

**DOI:** 10.1007/s40037-022-00729-5

**Published:** 2022-11-22

**Authors:** Parisa Rezaiefar, Yara Abou-Hamde, Farah Naz, Yasmine S. Alborhamy, Kori A. LaDonna

**Affiliations:** 1 Bruyère Academic Family Health Unit, Ottawa, Ontario Canada; 2 Department of Family Medicine, University of Ottawa, Ottawa, Ontario Canada; 3 Faculty of Education, University of Ottawa, Ottawa, Ontario Canada; 4 Department of Innovation in Medical Education, University of Ottawa, Ottawa, Ontario Canada; 5 Department of Medicine, University of Ottawa, Ottawa, Ontario Canada

**Keywords:** Underrepresented women, Medical training, Family Medicine, Medical education

## Abstract

**Introduction:**

Medicine remains an inequitable profession for women. Challenges are compounded for underrepresented women in medicine (UWiM), yet the complex features of underrepresentation and how they influence women’s career paths remain underexplored. This qualitative study examined the experiences of trainees self-identifying as UWiM, including how navigating underrepresentation influenced their envisioned career paths.

**Methods:**

Ten UWiM family medicine trainees from one Canadian institution participated in semi-structured group interviews. Thematic analysis of the data was informed by feminist epistemology and unfolded during an iterative process of data familiarization, coding, and theme generation.

**Results:**

Participants identified as UWiM based on visible and invisible identity markers. All participants experienced discrimination and “otherness”, but experiences differed based on how identities intersected. Participants spent considerable energy anticipating discrimination, navigating otherness, and assuming protective behaviours against real and perceived threats. Both altruism and a desire for personal safety and inclusion influenced their envisioned careers serving marginalized populations and mentoring underrepresented trainees.

**Discussion:**

Equity, diversity, and inclusion initiatives in medical education risk being of little value without a comprehensive and intersectional understanding of the visible and invisible identities of underrepresented trainees. UWiM trainees’ accounts suggest that they experience significant identity dissonance that may result in unintended consequences if left unaddressed. Our study generated the critical awareness required for medical educators and institutions to examine their biases and meet their obligation of creating a safer and more equitable environment for UWiM trainees.

**Supplementary Information:**

The online version of this article (10.1007/s40037-022-00729-5) contains supplementary material, which is available to authorized users.

## Introduction

Despite growing—and in some cases equal—representation of women in medicine across the Western world [1], women continue to face significant sociocultural and structural impediments to the advancement of their careers. Compared with their male counterparts, women experience lower job satisfaction, higher rates of burnout, and wage inequity in medicine [2–4]. Globally, women physicians are also less likely to pursue academic careers, attain leadership positions, or be promoted [5–8]. Inequity is rarely a single-issue phenomenon, however. Evidence suggests that such challenges are compounded for underrepresented women in medicine (UWiM). For example, although the overall proportion of full-time women faculty in US medical schools has increased steadily between 2009 and 2018, the percentage of women from a race or ethnicity considered to be underrepresented has stagnated at 13% [6].

Recent equity, diversity, and inclusion efforts have not eased these challenges, in part because efforts do not attend to the complexities of what being underrepresented in medicine (URiM) entails [9]. For instance, conceptualizations of “underrepresented” vary widely across institutions [10]. Some adopt the Association of American Medical Colleges definition verbatim, conceptualizing URiM as “racial and ethnic populations that are underrepresented in the medical profession relative to their numbers in the general population.” [11]. Others interpret URiM to include “individuals whose personal characteristics, such as race, ethnicity, socioeconomic status, health condition, or disability, place them at risk for conscious or subconscious bias or discrimination” [10]. In Canada, the Association of Faculties of Medicine of Canada (AFMC) does not have a formal definition of URiM. Nevertheless, AFMC leadership developed “the Equity and Diversity Audit Tool for Canadian Medical Schools” to help programs gauge how they are supporting designated equity groups, including Aboriginal peoples, women, persons with disabilities, and visible minorities [12].

More expansive definitions of URiM nod to the long-recognized complexities of navigating both visible and invisible markers [13], that have, so far, been overlooked and underexplored in the medical education context. However, to be truly meaningful, definitions of underrepresentation must also account for how having multiple visible and invisible underrepresented identities may compound marginalization for those learning and working in a historically male-dominant, heteronormative, and Euro-centric profession [14, 15]. In 1989, Kimberlé Crenshaw, a feminist critical race scholar, introduced the term *intersectionality* to account for the fact that, because of the intersection of their gender and race, Black women experience greater discrimination than both white women and Black men [16]. Approaching gender, race, or ethnicity as monolithic identities divorced from social and institutional power risks failing to effectively support those who may be located both “within overlapping systems of subordination and at the margins of feminism and antiracism” [16].

Unless they have a fulsome understanding of the meaning and impact of being UWiM, institutions and individual medical educators will struggle to support the personal and professional needs of UWiM trainees. Medical education scholars are increasingly calling for nuance, yet the complexities of underrepresentation remain underexplored [17, 18]. So far, the little we know focuses on quantifying the scope of racism or sexism [19, 20]; few studies have elucidated *how* such experiences of underrepresentation influence UWiMs’ perception of themselves as physicians [21–24]. Since equity, diversity, and inclusion work depends on such an understanding, we conducted a qualitative exploration to investigate how women trainees conceptualized underrepresentation in medicine and how their experiences influenced their envisioned careers.

## Methods

All study procedures were approved by Institutional Research Ethics Boards at two academic institutions affiliated with the training program (Protocols # H-05-20-5708 and # M16-20-036).

### Recruitment

Recruitment took place between June and September 2020. Using a departmental list, YAH and FN sent a recruitment email to all trainees (*n* = 159) enrolled in a family medicine postgraduate training program in Canada. Given the lack of consensus around definitions and the dearth of data on medical trainee demographics in Canada [25], we elected not to define underrepresentation. Instead, we invited participants to self-identify as UWiM based not only on race or ethnicity, but also on characteristics such as gender identity, sexuality, religion, age, physical build, disability (visible or invisible), or socioeconomic status (SES).

### Data collection

We collected data using group interviews, a method where “participants feed off of each other’s perceptions”[26, p. 100] to generate rich data. Group interviews also facilitated both the scholarly work of YAH and FN and the participation of UWiM trainees during the COVID-19 pandemic—a global emergency disproportionately affecting women, particularly those identifying as underrepresented. Specifically, adding group interviews at the end of trainees’ academic day was an ideal method of collecting multiple perspectives with minimum interruption to their clinical duties.

Ten trainees consented to participate in one of three group interviews led by YAH. Because of COVID-19 restrictions, group interviews were conducted using GoToMeeting. Each interview was attended by 2–5 participants and lasted between 45 and 140 min. Using a semi-structured guide, YAH posed the following overarching questions to guide conversations:

1. What defines you as UWiM?
2. How has your UWiM identity influenced your training experience thus far?
3. How has your UWiM identity impacted your interactions with patients and other members of the healthcare team?
4. How do you perceive your experiences as a UWiM influence your professional or career choices?

All group interviews were audio-recorded and transcribed using NVivo automated transcription software [27]. YAH and FN ensured the accuracy and anonymization of transcripts.

### Data analysis

Guided by Braun and Clarke’s [28] six-phased approach to reflexive thematic analysis, team members independently read transcripts to familiarize themselves with the data. Next, YSA engaged in selective coding to identify preliminary themes that were refined and finalized over a series of team meetings. Finally, YAH and YSA re-coded the entire dataset. Throughout the analytical process, team members identified data patterns, constructing increasingly interpretive analytical ‘storylines’ aimed at understanding participants’ experiences of underrepresentation in medicine.

Data analysis was informed using the feminist epistemology described by Anderson, which centralizes participants’ perspectives [29]. Although we did not use an *a priori* theoretical framework to deductively analyze the data, we were sensitized to critical race theory and intersectionality [16, 30, 31]. Specifically, we attended to how participants conceptualized their identities, how they perceived their identities influenced their training experiences, and how navigating such experiences affected their self-perceptions as professionals and their envisioned future practice.

### Reflexivity

Reflexivity—a process of thoughtfully considering how each researcher’s personal and professional experiences might influence the analytical process—is a key component of qualitative rigor [32]. We have included a rich reflexive account in Table 1 of the Electronic Supplementary Material.

## Results

**Fig. 1:**
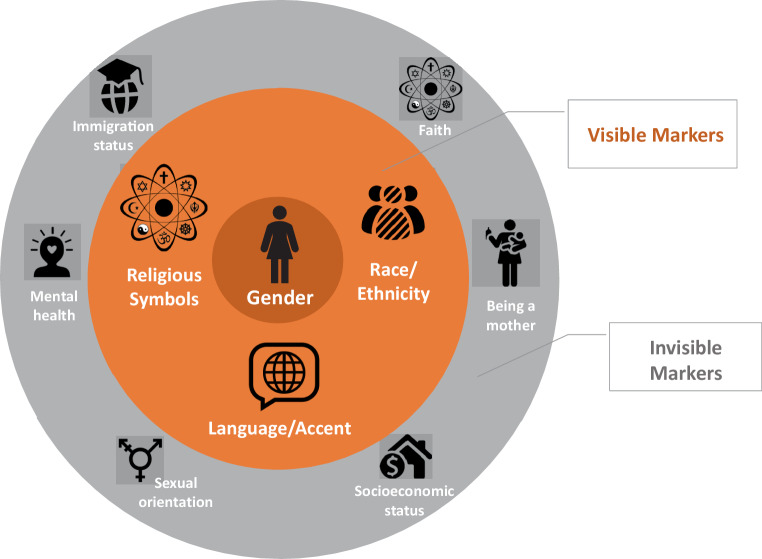
Descriptive analysis of underrepresented identities as reported by women. In addition to **gender**, each participant endorsed having at least one visible (*orange*) and/or invisible (*grey*) identity marker

Participants identified as being UWiM based on a diverse range of identities (Fig. 1). Half of participants were international medical graduates and half reported English as their first language. Eight participants reported being a member of a visible minority because of race, ethnicity (sometimes inferred from one’s name or accent), or religious symbols such as the hijab. Other invisible identities that contributed to feeling marginalized included sexual orientation, religion, low SES, mental health, immigration status, and motherhood. To protect their anonymity, we have not provided additional descriptive details about participants’ often-intersecting identities.

Participants’ identities, whether visible or invisible, resulted in a universal self-perception that they were different from what was familiar, expected, or generally accepted across the profession—a concept defined as otherness [33]. However, the experience and implications of otherness appeared to differ depending on whether participants’ identity markers were visible or not. For instance, those who were racialized or who wore a religious symbol were more likely to report overt instances of discrimination:

“I’ve had patients refuse care from me at (two local hospitals) because I’m a person of colour. And that’s taken away learning opportunities and made for really awkward staff interactions who don’t know how to deal with that on the floor.” (P1, GI1)

Conversely, those with less readily apparent identity markers such as ethnicity, sexual orientation, or low SES were more likely to witness intolerant behaviour rather than be directly targeted by it. For example, a participant who did not “*read as visibly*” (P9, GI3) of mixed race witnessed anti-Chinese sentiment from colleagues in the context of the coronavirus pandemic. She encapsulated the harm caused by being simultaneously othered and outwardly belonging by noting:

“It’s interesting that having those identities being more hidden has caused a lot of people to make certain comments or certain jokes, assuming I’m going to laugh or go along with it, when in reality, that’s really hurtful or offensive to me.” (P9, GI3)

Otherness resulted not only from overt discrimination in clinical workplaces, but also from prior life experiences, which primed participants to assume that anyone could be a potential threat. One participant identifying as lesbian described that, although she had not had a traumatic encounter with a patient, the pervasiveness of homophobia meant that she had to assume anyone could cause her harm. She ruminated on when and how to hide aspects of her personal life that might make her a target:

“I am doing those calculations with every patient interaction … Will [talking about my wife] affect how they think of me? It’s just the mental work that you’re doing in the back of your mind.” (P7, GI2)

Indeed, all participants spent considerable time and energy both anticipating discrimination and assuming defensive behaviours against real or perceived threats. Since many participants expressed apprehension about disclosing religious beliefs in clinical environments, religion is a key example illustrating how the visibility or invisibility of identity markers variably affected participants’ abilities to maneuver potentially harmful experiences. For example, like Participant 7 who responded to potential homophobia by keeping her sexual orientation private, Participant 4 refrained, with great regret, from advocating for a patient because she feared that revealing shared religious beliefs would bias perceptions about her competence:

“[A patient] refused the DNR simply because she said that she’s a Roman Catholic. The senior staff doctor and the nurses were making fun of that in front of me. I just shut my mouth. Didn’t say anything because if you say something, they might say, Oh, how would you function as a competent doctor if you let your religion or emotion lead you in a direction or another.” (P4, GI1)

Such strategic identity suppression was more complicated for those with visible minority status. In sharing their experiences, participants realized that the ability to conceal aspects of one’s identity, though disempowering, was simultaneously a privilege not universally available to all UWiM. For Participant 7 who was white, hiding her sexuality meant she could conceal her UWiM status entirely—an impossibility for a marginalized Muslim participant who wondered whether ceasing to wear hijab might help:

“I am more Western-minded but more ethnic-looking [because of the hijab]. Is it a better idea if I sort of remove the look that makes me look very obviously Muslim, because some people will be Islamophobic? I don’t want that to stand in my way for my career.” (P10, GI3)

For Participants 4 and 10, who reported that their ethnicities and accents made them stand out, removing a visible religious symbol or concealing a religious affiliation were only partial solutions for shielding against workplace discrimination. Although all participants were privy to gender-based discrimination, the burden of navigating otherness was magnified for members of visible minority groups:

“The difference that you [Participant 7] highlighted between our experiences is that you can almost hide the part of your identity that may become a challenge, right. Whereas for me it’s like, hello, I’m Black. Everybody knows I’m Black. So, I think it must be incredibly stressful for you to feel like you’re walking on eggshells around the subject of sexual identity and that at any moment somebody might say something inappropriate. Whereas for me, I feel like everybody around me is walking on eggshells and trying not to say the wrong thing about Blackness. And I’m just waiting for someone to say the wrong things.” (P6, GI2)

Participants’ experiences of discrimination and otherness were exacerbated by the absence of faculty support. Notably, no participant shared an example of a faculty member who advocated on her behalf. Worse, faculty members perpetuated discrimination by failing to use their authority to intervene:

“The ones that ignore it are the most frustrating. The ones that think it’s okay for me to walk into a room, walk out with tears in my eyes, and then just [say] ‘don’t worry about it, I’ll see that patient then.’ That’s not helpful. You not acknowledging the fact that I’ve gone through that abuse is not helpful.” (P1, GI1)

Experiences of discrimination, otherness, and the lack of faculty advocacy profoundly influenced how participants conceptualized their professional paths. Most envisioned a career path aimed at creating safer, more inclusive clinical and learning environments. For instance, a participant who struggled with mental health was motivated to de-stigmatize personal vulnerabilities for her future trainees:

“If you have anything going on with mental health, it’s really difficult to tread the line between help seeking and coming off as a person who’s in distress. I know I’m going to be staff very soon. I think it’s something that I try and say when I meet new residents … ‘If you’re feeling stressed, let me know’.” (P2, GI1)

Additionally, participants expressed a strong desire to serve vulnerable patient populations—perceiving that while individual experiences of discrimination may be unique, the shared hardships of being marginalized established common ground:

“I really love inner city health and find addictions medicine interesting. I think refugee health is very interesting. And these are all areas that don’t affect me … But because of my experiences, I feel like I can sort of stretch my mind to understand and empathize with their experiences as well.” (P6, GI2)

However, although participants emerged from lived adversity empowered to teach and practice medicine with empathy and altruism, their career choices also seemed motivated by a need for personal safety, aiming to carve out a professional space where they could insulate themselves from discrimination. For instance, Participant 7 contrasted her current reality with the future she envisioned working with adolescents and LGBTQ+ patients: “*I’m drawn to the adolescent population*” (P7, GI2). Specifically, she believed “*finding your allies or people who identify like you*” (P7, GI2) would empower her to practice in an authentic and inclusive way not only for patients but also for herself. Participant 3 expressed a similar desire for safety: “*I just want to go to an environment where I’m around like-minded people and don’t have to stress about this [overt discrimination]*” (P3, GI1).

## Discussion

UWiM interviewees described a variety of identity markers that made them feel othered during their training that are not always conceptualized as facets of underrepresentation in current definitions. Participants’ accounts shed light not only on the frequency of overt and covert discrimination UWiM trainees endure, but also on the considerable cognitive and emotional labor they expend to anticipate and deflect harms perpetrated in academic environments. Our study provides a much-needed starting point for understanding how otherness and discrimination may make the medical education environment oppressive and hostile for UWiM.

Participants’ accounts demonstrate how power and privilege force subjugated populations to think and act through lenses that members of dominant groups impose on them. In response and as a survival mechanism to oppression, members of minority groups are forced to redefine their identity through the lens of the dominant culture, a phenomenon described by Du Bois [34] in 1903 in the context of African Americans and further developed as “triple consciousness” by Deborah Gray White to include racialized women. Though in the Canadian context and not solely focused on Black women, our participants’ experiences resonates with triple consciousness. Study participants maneuvered their multiple, ‘othered’ identities by engaging in well-known trauma responses [35], including hypervigilance or “excessive scanning” [36] of their learning environment for potential threats, and by concealing aspects of their identities to either facilitate belonging or to avoid overt discrimination [37–39]. Full identity concealment was noted as a form of privilege, however. Thus, although white women who identify as underrepresented are certainly harmed by workplace discrimination, their whiteness affords “an invisible weightless knapsack of special provisions, maps, passports, codebooks, visas, clothes, tools and blank checks” that ease the burden of navigating intersectionality [40].

Nevertheless, participants’ accounts revealed both the trauma of discrimination and the emotional labor spent navigating otherness in clinical learning environments [41, 42]. While managing the typical stresses of residency training, participants’ learning attention and emotional energy were constantly diverted by signals that they may not fit who they are *supposed* to be as doctors, particularly in academic settings where power, connotated by leadership and academic rank, remains mostly in the hands of white men and, to a lesser extent, white women [6, 43–45]. Participants’ experiences resonated with those published recently by Monnique Johnson in an editorial explicating the toll of being a Black medical student in primarily white training environments: “For me, my whole being when I had to interact in these spaces felt like a uniform or a mask. Exhaustion came from not having a space to be unapologetically and authentically myself and not having enough people who could relate to me” [46].

Testimonies by participants, Ms. Johnson, and others raise questions about the equity of learning experiences and structures. Institutional leaders and medical educators must recognize that underrepresented trainees experience significant identity dissonance during their training, often in silence and in silos.

We must also be mindful that medicine does not provide adequate space for trainees who identify as URiM or UWiM to heal from the trauma of discrimination [47]. Unsupported, participants were left alone to manage the tension between their personal and idealized professional identities, making decisions rooted in fear and exclusion that could have life-long personal and career consequences. Safe spaces—under guidance from trusted mentors—are required for UWiM trainees to debrief when encountering discrimination and to reflect on the impact of such experiences on their professional identity and intended career choices. Although women, particularly those who are underrepresented, need skilled and compassionate mentors to nurture their careers, they more so need *advocates *who are willing to both call out discrimination when it happens, and to take meaningful action to dismantle the discriminatory forces that impact women both personally and professionally.

In the absence of faculty advocates, participants appeared driven to correct the harms—including blatant discrimination and problematic inaction—that they experienced. They envisioned a career path where they could provide the mentoring, advocacy, and opportunity to be authentically human that they were denied. Although it is admirable to emerge from such dissonance with a sense of service, participants seemed unaware of potential drawbacks. For example, caring for populations with complex psychosocial and health care needs is time-intensive work that risks exacerbating compassion fatigue and burnout—phenomena that are already well-documented among female physicians.

The desire to serve marginalized populations may be another possible explanation for the paucity of UWiM in academia and leadership. That is, although discriminatory structural issues are known to create a “leaky pipeline” [47] that hinders women’s ability to attain leadership positions, the trauma response to inequities might lead to self-exclusion from positions of power. Although participants seemed genuinely excited and empowered to embark on a career path that would improve training and care for marginalized patients and trainees, *all *physicians should have the skillset and motivation to do this work. These are fundamental components of being a culturally competent physician, yet UWiM participants seemed to perceive them as part of a specialized career path—a problematic notion that should trigger both reflection and action.

### Limitations

Experiences of otherness and discrimination are uniquely harmful, rendering it impossible for a qualitative dataset that is aimed at exploring these harms to become “*saturated*” [48]. We acknowledge that a sample of 10 women from one Canadian academic institution, who may have withheld information they did not feel comfortable sharing in a group setting, generates an incomplete understanding of otherness in medicine. However, participants’ rich and emotive accounts provided invaluable nuance for understanding the complexities that UWiM must navigate. Because qualitative findings are intended to be transferable rather than generalizable, findings may resonate with trainees and faculty members across gender identities and practice settings. More research is urgently needed, and we are currently embarking on a national study to understand the relationship between intersectionality and the professional identity formation of UWiM.

## Conclusion

For many trainees, overt discrimination and feelings of otherness are ever-present threats in academic environments. Policies and individual actions aimed at equity, diversity, and inclusion must consider the complexities of underrepresentation that exacerbate oppression. Faculties of medicine would benefit from a thoughtful approach to conceptualizing underrepresentation in medicine, taking local and historical contexts into account. Ensuring that definitions and policies are equitable and inclusive depends on both a comprehensive understanding of the breadth of visible and invisible identities represented by trainees and on stratifying demographics data to ensure that any evolving conceptualizations of underrepresentation in medicine do not dilute efforts aimed at supporting historically minoritized groups [11]. Postgraduate programs and individual educators have a duty not only to ensure that training environments are culturally safe but also that they foster “the pedagogical space to facilitate sense-making processes … thereby enabling [trainees] to understand their own developing identities as doctors” [49]. We urge the medical education community to heed Dr. Maya Angelou’s instruction to “do the best you can until you know better. Then when you know better, do better” [50]. Our participants generated the critical awareness necessary for faculty to “do better” by using their power and privilege to advocate for an inclusive learning environment where all trainees can thrive.

## Supplementary Information

Table 1 Reflexivity
