# Supplementary material for: “Walking on eggshells”: experiences of underrepresented women in medical training
Source: Perspect Med Educ. 2022 Nov 22;11(6):325–32. doi: 10.1007/s40037-022-00729-5 (PMC9684928; doi:10.1007/s40037-022-00729-5)
Supplement: Supplementary file 1 — Table 1 Reflexivity [file 40037_2022_729_MOESM1_ESM.docx]

| **Table 1.** Reflexivity | |
| --- | --- |
| **Name** | **Reflexivity** |
| PR | The principal investigator of this study is a heterosexual cis-woman and a family physician. She identifies as a UWiM because of her ethnicity, mother tongue, religion, poverty experience, and being the first woman to attain a post-secondary degree in her family. PR’s engagement with the data was influenced by her 20 years of clinician experience with special interest in vulnerable patient populations. As a clinician-educator, she has supervised close to 100 family medicine trainees in academia. As a UWiM faculty member, she has experienced and witnessed discrimination. This led to viewing the data through the lens of a faculty member wanting to advocate for trainees, yet often unable to do so effectively because of her UWiM status and other systemic factors. |
| YA | She was a family medicine trainee at the time of this study. She considers herself UWiM because of her refugee and immigrant experience, ethnicity, mother tongue, and her family’s low SES. To data generation and analysis, she brought the lens of a trainee who often felt compelled to hide aspects of her personal identity in clinical and educational settings but who also viewed her minority experiences as a privilege in caring for vulnerable patients. |
| FN | She was a family medicine and international medical graduate trainee at the time of this study. She identifies as a cis-woman and UWiM because of her skin color, place of birth, the religious symbol she wears, and her mother tongue. As two UWiM family medicine trainees, YA and FN played a crucial role in mitigating power imbalances between researchers and participants. |
| YSA | She is a first-generation immigrant and a mother who received most of her education (except for her MA which she obtained in Canada) in her country of birth. She self-identifies as a heterosexual cis woman who is UWiM because of her place of birth, mother tongue, and the religious symbol she wears. Although she is not a medical trainee, she is able to relate to the discrimination experiences of participants and the feeling of being “othered,” and the data were eye opening to what people can go through silently and invisibly. |
| KAL | She is a PhD scientist, an expert qualitative research methodologist, and a mother. Although she is an immigrant, she is aware that her identity as a white, heterosexual cis woman who speaks English as a first language shields her from the discrimination and xenophobia experienced by those who are underrepresented in both medicine and North America more broadly. Since medical education is a woman-dominant field, KAL does not identify as being othered in her professional work. KAL’s view of the data was informed by her research exploring the hidden or overlooked struggles of medical trainees and faculty, including burnout and workplace discrimination. |
